# Case Report: A rare presentation of gastrointestinal amyloidosis: unmasking the hidden culprit of chronic epigastric pain and weight loss

**DOI:** 10.3389/fgstr.2026.1742374

**Published:** 2026-04-13

**Authors:** FNU Veena, Muhammad Rizwan Akram, Mohamed Farag, Priscilla Lajara Hallal, Elona Shehi

**Affiliations:** Department of Internal Medicine, BronxCare Health System, New York, NY, United States

**Keywords:** AL amyloidosis, chronic epigastric pain, Congo red staining, hepatitis B, monoclonal gammopathy, weight loss

## Abstract

Amyloidosis is a rare, heterogeneous condition characterized by extracellular deposition of misfolded protein fibrils, resulting in organ dysfunction. Gastrointestinal amyloidosis (GIA), an uncommon manifestation, is often underdiagnosed due to its nonspecific symptoms, such as weight loss, abdominal pain, and diarrhea. Early recognition and accurate amyloid typing are crucial, as treatment strategies depend on the specific subtype involved. We report a case of a 42-year-old African male presenting with chronic epigastric pain, significant weight loss (BMI: 17.1), and a history of GERD. He had recently traveled to Equatorial Guinea. His symptoms, including nausea, decreased appetite, constipation, and persistent epigastric pain, were unresponsive to proton pump inhibitors. On examination, he was hypotensive and tachycardic. Laboratory workup revealed hyponatremia and elevated troponin T. Imaging showed fecal impaction and pyloric edema. Upper endoscopy and colonoscopy with biopsies confirmed amyloid deposition in the stomach, duodenum, ileum, and colon. Congo red staining demonstrated classic apple-green birefringence under polarized light. Cardiac evaluation revealed reduced global longitudinal strain. Serum studies showed elevated free lambda light chains and an IgG-lambda monoclonal band, while the urine protein/creatinine ratio was 361 mg/g, consistent with proteinuria. Chronic hepatitis B infection with detectable HBV DNA and elevated gamma/delta T cells on the lymphoma panel raised concerns for an underlying T-cell lymphoproliferative disorder. This case highlights the diagnostic complexity of gastrointestinal amyloidosis with multisystem involvement. The patient’s presentation, along with elevated free light chains and monoclonal gammopathy, raised suspicion for AL amyloidosis, though chronic hepatitis B and possible lymphoproliferative disease kept AA amyloidosis in the differential. Consistent with the literature, vague gastrointestinal (GI) symptoms often delay diagnosis, underscoring the role of Congo red staining and mass spectrometry for confirmation and subtype identification. Recent trials support daratumumab for AL and patisiran/inotersen for ATTR amyloidosis. Early, accurate diagnosis is key to initiating appropriate therapy and improving outcomes, with multidisciplinary involvement crucial for optimal care.

## Introduction

Gastrointestinal amyloidosis (GIA) is a complex protein deposition condition that poses considerable diagnostic and therapeutic difficulties (1). This arises from the aggregation of insoluble extracellular protein fragments in multiple organs of the gastrointestinal tract, resulting in structural and functional deterioration (1). Although gastrointestinal involvement is prevalent in systemic amyloidosis, symptomatic instances with biopsy-confirmed evidence are comparatively uncommon (2).

The clinical manifestation of GIA is diverse, with prevalent symptoms including stomach pain, weight loss, diarrhea, and gastrointestinal hemorrhage (2). These symptoms frequently result in a significant delay in diagnosis, with a median duration of 7 months reported in certain investigations (2).

Symptomatic GIA with biopsy-confirmed specimens is relatively rare, with findings ranging from normal mucosa to ulcerations (3). Nonetheless, gastrointestinal (GI) amyloidosis frequently indicates systemic involvement, especially cardiac amyloidosis in light chain amyloid (AL) and transthyretin amyloidosis (ATTR) types, highlighting the necessity for thorough assessment in these individuals (2).

This case report describes a 42-year-old male suffering from severe epigastric pain and weight loss, who was ultimately diagnosed with gastrointestinal amyloidosis. This example illustrates the difficulties in detecting this disorder and underscores the necessity of considering amyloidosis in patients presenting with unexplained gastrointestinal complaints, particularly when associated with unintended weight loss (4).

## Case presentation

A 42-year-old African male with a BMI of 17.1 and a history of gastroesophageal reflux disease presented to the emergency department with chronic epigastric pain and unintentional weight loss. The patient had recently returned from Equatorial Guinea and had been experiencing persistent epigastric pain unresponsive to proton pump inhibitor therapy for nearly a year. Other associated symptoms included nausea, decreased appetite, and constipation. In addition to denying a history of cancer, HIV, or lymphoma, he also denied having recent melena, hematemesis, or hematochezia.

The patient was alert and oriented on examination, but demonstrated hypotension (BP 62/42 mmHg), tachycardia (HR 130 bpm), and positive orthostatic hypotension. Preliminary test results revealed mild hyponatremia (132 mEq/L) and elevated troponin T levels (34 ng/L). **Table 1** shows the patient’s laboratory findings. The EKG demonstrated sinus tachycardia with T wave inversions in leads II, III, aVF, and V3–V4.

**Table 1:** Laboratory values.

Lab test	Result	Reference value
WBC Count	6.5	[4.8-10.8 k/ul]
RBC Count	4.58	[4.50-5.90 MIL/ul]
HGB	13.7	[12.0-16.0 g/dl]
Hematocrit, Whole Blood	40.5	[42.0-51.0%]
MCV	88.4	[80.0-96.0 fL]
RDW	14.4	[10.5-14.5%]
Platelet	248	[150–400 k/ul]
Sodium	132	[135–145 mEq/L]
Potassium	3.9	[3.5–5 mEq/L]
Chloride	98	[98–108 mEq/L]
Glucose	96	[70–120 mg/dl]
BUN	9.0	8.0-26.0 mg/dl]
Creatinine	0.8	[0.5-1.5 mg/dl]
Iron, Serum	**47**	[65–175 ug/dl]
Ferritin, Serum	**326.0**	[13.0-150.0 ng/mL]
Unsaturated Iron Binding Capacity	124	[112–346 ug/dl]
Creatine Kinase, Serum	50	[20–200 unit/L]
Troponin T, Serum	**34**	[<=12 ng/L]
HIV ½ O Ab	Nonreactive	
Urine Protein	24	0–31 mg/dl
Free Lamda Serum	**208.5**	5.7-26.3 mg/L
Free Kappa Serum	**20.4**	3.3-19.4 mg/L
Urine Protein/Creat. Ratio (mg/g creat)	**361**	25-148 (mg/g creat)
Hepatitis B Surface Antigen	**Reactive**	
Hepatitis B Surface Antibody	Non-Reactive	
Hepatitis B Core IgM Antibody	Non-reactive	
Hepatitis B Core Total Antibody	**Reactive**	
Hepatitis Be Antibody	**Reactive**	
Hepatitis Be Antigen	Non-Reactive	
Hepatitis B Vir DNA IU/Ml	**180 [IU/mL]**	
Hepatitis B Vir DNA Log IU/ML	**2.26 [Log IU/mL]**	

Values in bold are outside of the normal range.

**Figure 1:**
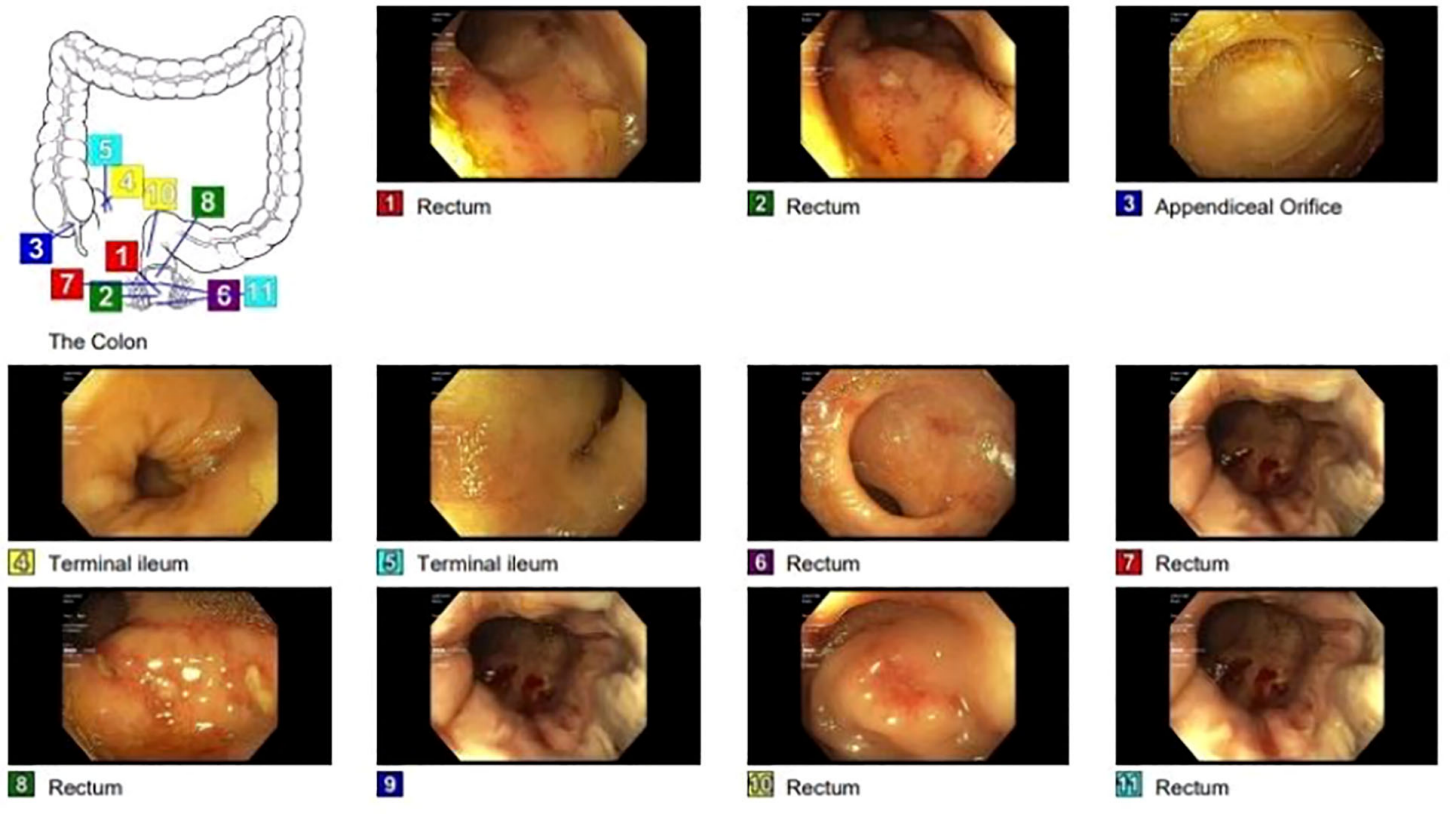
Colonoscopy shows localized moderate mucosal changes characterized by congestion (edema), erosions, mucus, and confluent ulcerations in the distal rectum.

A CT scan of the abdomen indicated substantial colorectal fecal impaction and edematous changes in the pyloric channel walls. Subsequent evaluation involved endoscopic interventions. Colonoscopy showed localized moderate mucosal changes characterized by congestion (edema), erosions, mucus, and confluent ulcerations in the distal rectum; random biopsies were obtained (**Figure 1**).

**Figure 2:**
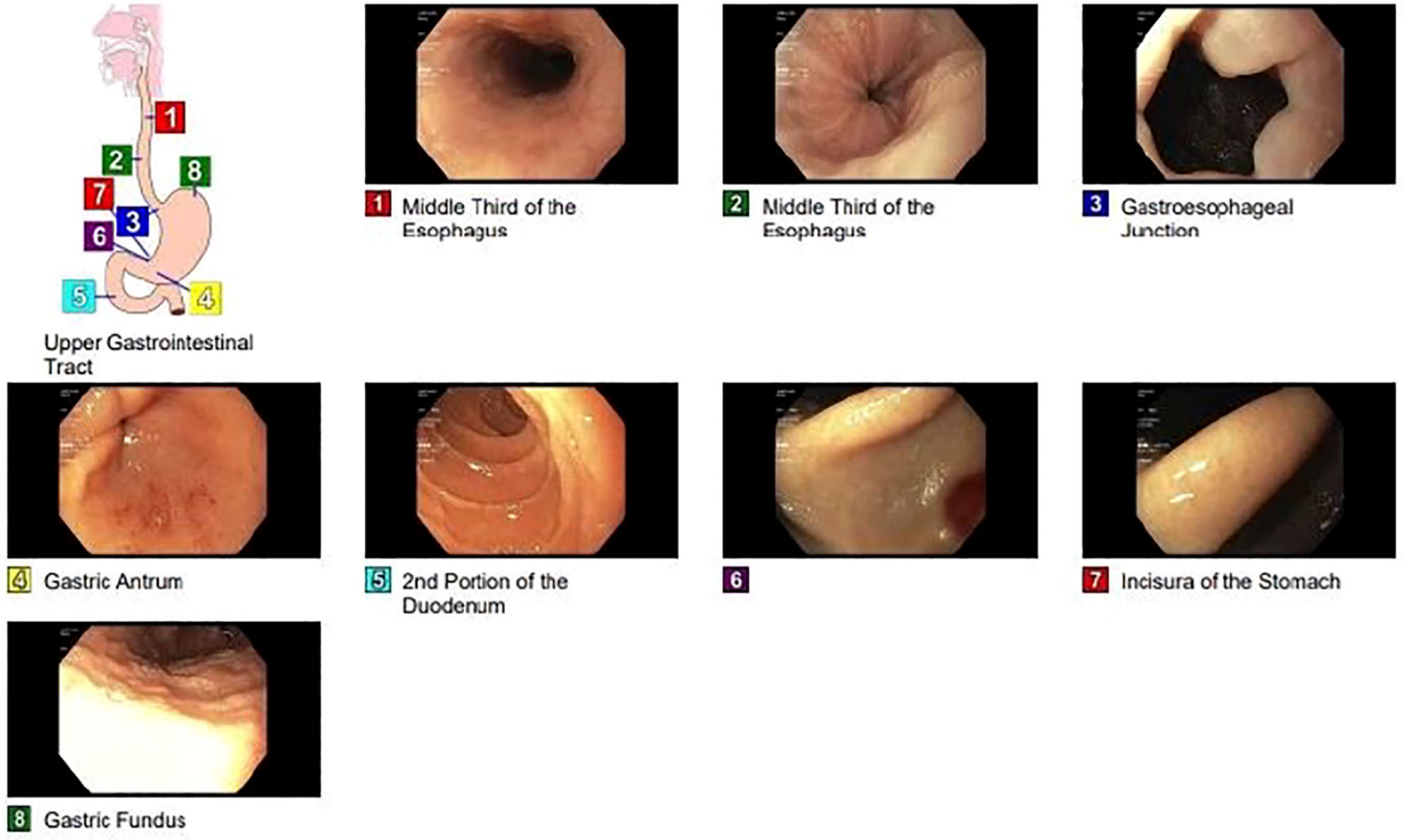
Upper gastrointestinal endoscopy shows erythematous mucosa in the antrum.

Upper endoscopy showed erythematous mucosa in the antrum. Biopsies were obtained from the duodenum and stomach (**Figure 2**).

**Figure 3:**
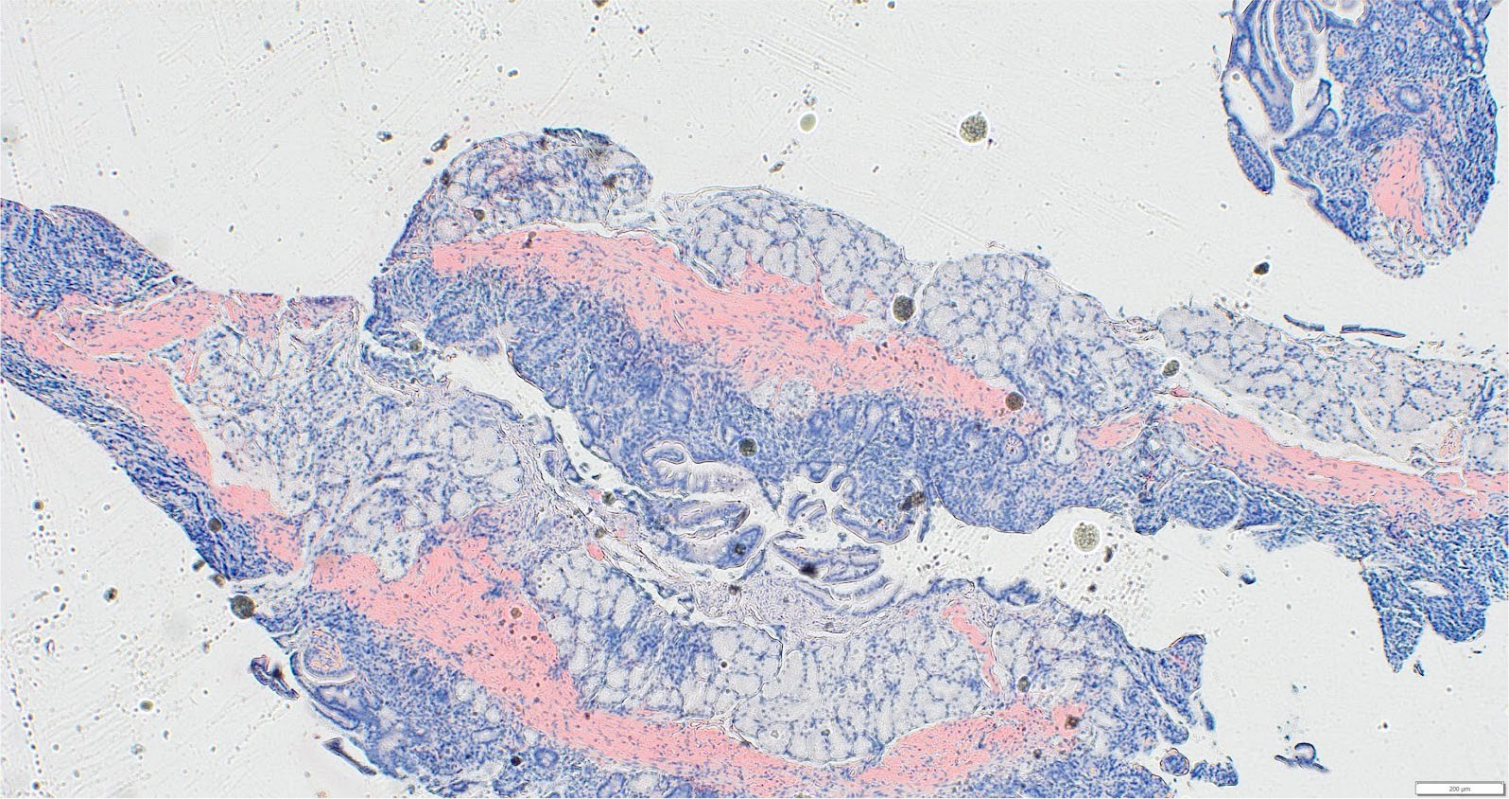
Small bowel with salmon-pink Congo red staining.

**Figure 4:**
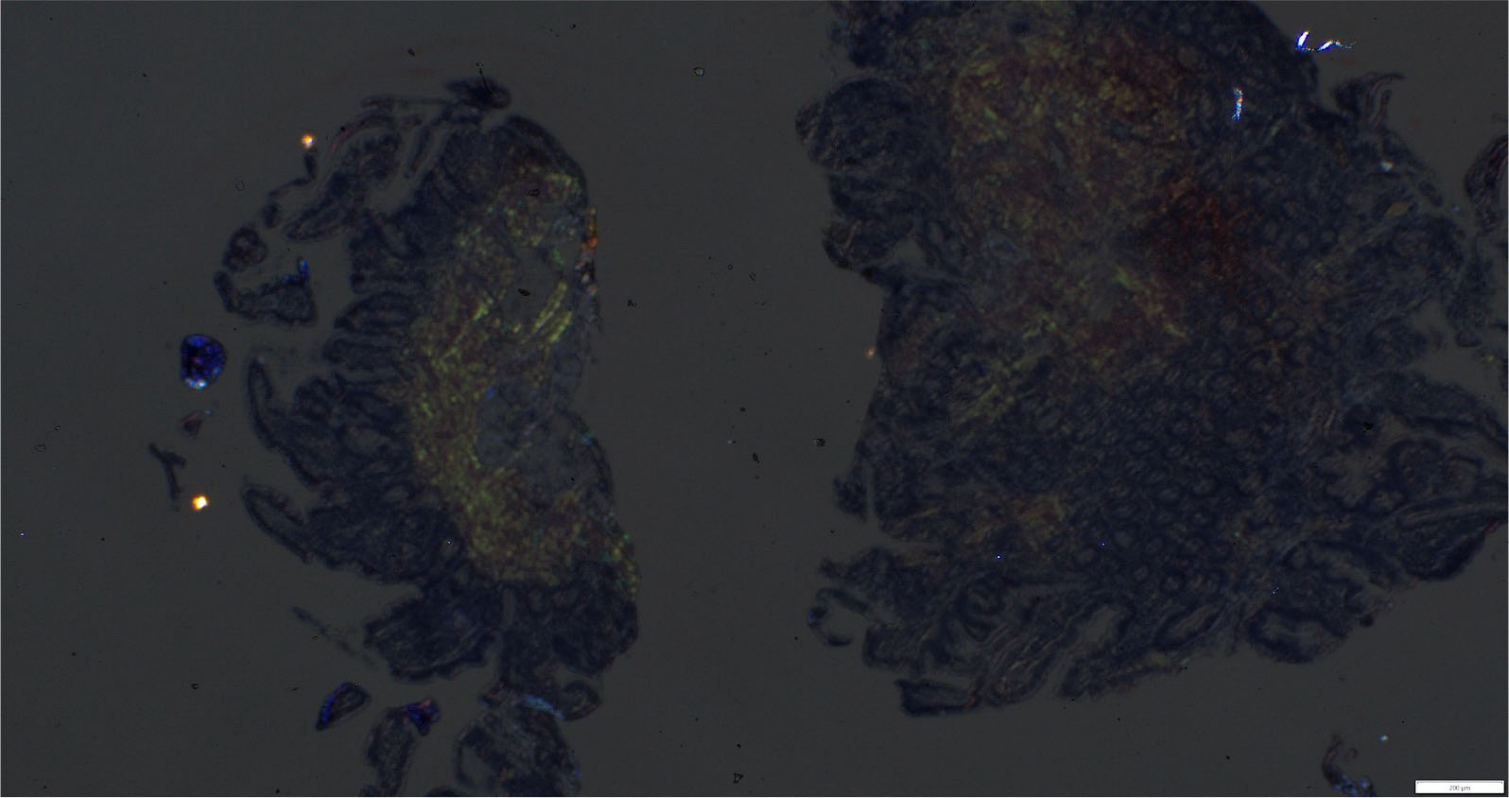
Small bowel showing apple-green birefringence with polarization.

**Figure 5:**
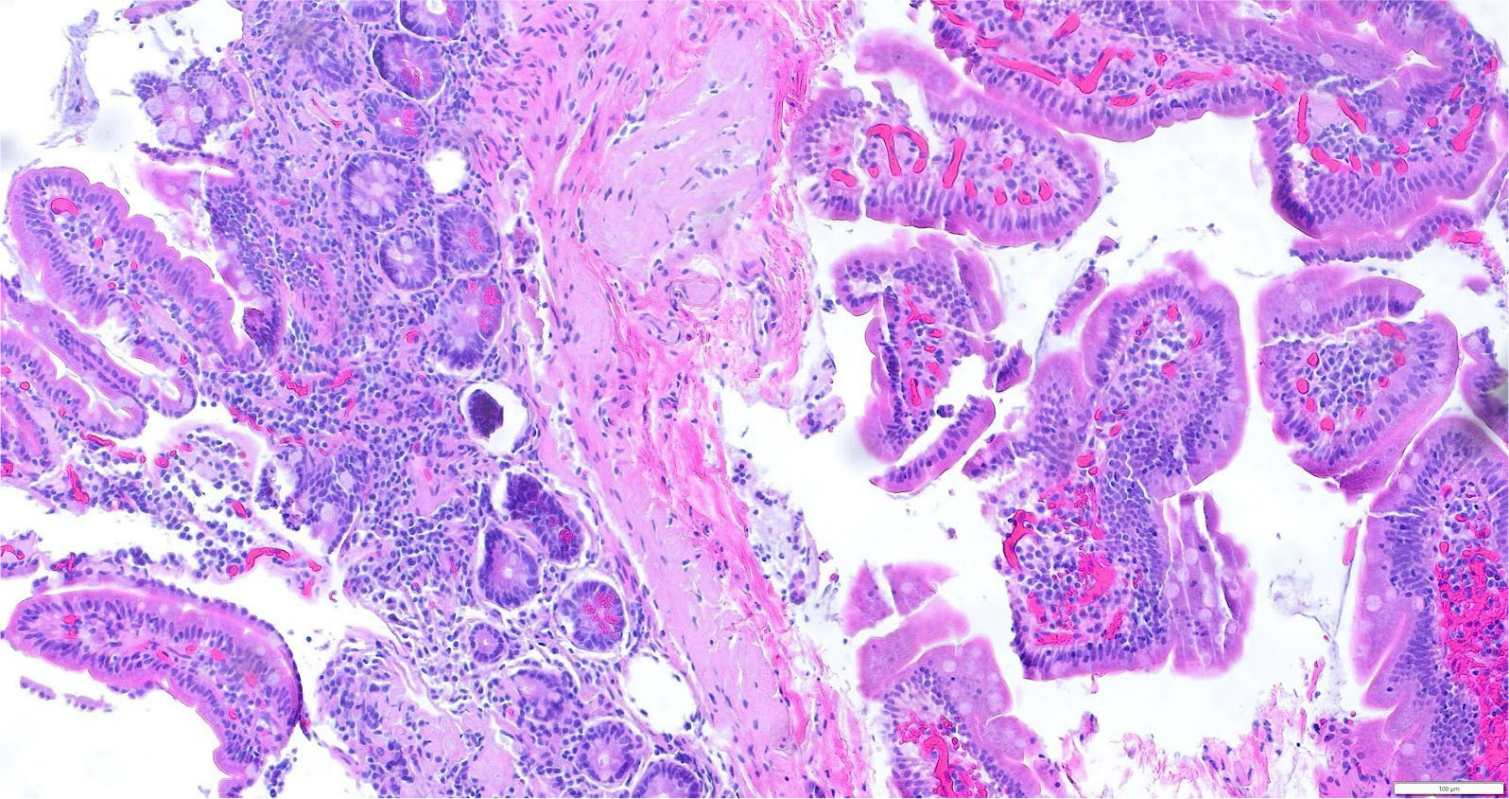
Small bowel showing waxy amyloid deposits on H&E staining.

Pathologic studies from both procedures indicated moderate chronic gastritis and background reactive changes in the stomach, and amyloidosis was found in the stomach, duodenum, terminal ileum, colon, and rectum (**Figures 3**-**5**).

Subsequent investigations included an echocardiogram that revealed preserved left ventricular ejection fraction (61.85%) but diminished global longitudinal strain (12.5%) with preserved apical segments, indicative of potential cardiac amyloidosis. Additional blood testing showed increased serum-free lambda and kappa light chains. Urine analysis revealed an elevated protein/creatinine ratio (361 mg/g creatinine) and a faint IgG-Lambda monoclonal protein band, along with a free monoclonal lambda band on immunofixation. The leukemia and lymphoma panel revealed elevated gamma/delta T cells (45% monotypic, 16% of total cells), suggesting possible T-cell lymphoma. The transthyretin (TTR) DNA sequence test was negative, ruling out familial amyloidosis.

Hematology was consulted, and evaluation for malignancy as an etiology included a leukemia/lymphoma panel that showed 45% of gamma/delta monotypic T cells. This was deemed most likely reactive. Further evaluation for lymphoma with CT abdomen and pelvis imaging found no organomegaly or adenopathy. CT head, CT neck, and CT chest with contrast were all unremarkable. The patient received supportive care with pain management, nutrition, and multidisciplinary follow-up.

## Discussion

Diagnosing and treating gastrointestinal amyloidosis poses a challenge, with ambiguous symptoms that can result in significant delays in making the correct diagnosis. The etiology of GIA is diverse, with AL amyloidosis as the most common variant, followed by ATTR and AA types (5). Secondary AA amyloidosis is the most prevalent in Africa (6). Deposits in AA amyloidosis accumulate due to the inflammatory secretion of the acute-phase reactant serum amyloid A (SAA) protein (7).

The significance of early identification and precise GI amyloidosis typing has been emphasized in recent studies. A proteomics investigation revealed 12 distinct amyloid types in gastrointestinal tissues, highlighting the necessity for reliable typing approaches, as treatment strategies rely on accurate identification of the amyloid type (5).

Moreover, red flags for GIA include early satiety, unexplained weight loss, chronic malabsorption, and diarrhea, GI bleeding, and pseudo-obstruction (8, 9). This patient presented with unexplained weight loss and epigastric pain, which warranted prompt investigation and management.

Recent research clarifies the frequency and features of GIA. AL amyloidosis remained the most prevalent type (77.9%), followed by ATTR (11.3%) and AA (6.6%), according to a thorough study of 2,511 GI amyloidosis specimens classified using proteomics-based techniques (5). This distribution emphasizes the need for correct typing, as the type of amyloid determines the course of therapy.

Different mutations have been associated with GI amyloidosis. These include Glu89Gln, Val30Met, Glu54Gln, and Gly47Glu TTR mutations (8). Our patient was negative for all mutations tested.

Our example illustrates the varied clinical presentation of GIA, which is in line with results from larger cohort studies. Sattianayagam et al. indicated that among patients with systemic amyloidosis, only 16.8% exhibited gastrointestinal signs and symptoms, of which fewer than half had biopsy-confirmed gastrointestinal amyloidosis (10). In patients with unexplained GI symptoms, this emphasizes the difficulty in detecting GIA and the need to keep a low threshold for performing biopsies with Congo red staining (5). The diagnosis of AL amyloidosis requires evidence of amyloid deposits in tissue (target or surrogate) and evidence of plasma cell dyscrasia. Tissue amyloid deposits show green birefringence when stained with Congo red dye and viewed with polarized light microscopy (11).

The clinical manifestations of systemic amyloidosis are primarily determined by the precursor protein and the involved organs. Nonetheless, there is considerable clinical overlap between all amyloidosis types. Commonly involved organs are the heart, kidneys, nervous system, liver, and gastrointestinal tract. The lungs, muscle, and soft tissue can also be affected. Some types typically cause symptoms in one predominant organ [i.e., wild-type transthyretin (ATTR) amyloidosis and the heart], whereas other types tend to present with multi-organ involvement [i.e., light-chain (AL) amyloidosis] (12, 13). The patient’s manifestation of persistent epigastric discomfort and weight loss corresponds with prevalent symptoms documented in the literature. Cowan et al. conducted research revealing that weight loss (43%), gastrointestinal bleeding (36%), and diarrhea (29%) were the most prevalent presenting symptoms in patients with GIA (14). The median duration from symptom onset to diagnosis was recorded as 7 months, highlighting the frequently extended diagnostic process.

Managing GIA is complex, necessitating therapeutic strategies customized to the specific amyloid type. High-dose chemotherapy followed by autologous stem cell transplantation has demonstrated the most promising outcomes for AL amyloidosis, which accounts for the majority of patients (15). However, this approach is not suitable for all patients, particularly those with advanced organ involvement.

Gastrointestinal amyloidomas can be treated with resection, chemotherapy, and symptomatic management. Historically, localized gastrointestinal amyloidomas have had an excellent prognosis. We also highlight that patients may be simply monitored over time if unable to pursue more definitive treatment (14–16). Given the rarity of the disease, there is no consensus or guideline on how to treat gastrointestinal tract amyloidomas. However, there is a large cure rate reported by surgical resection alone among those tumors that are resectable, with an immediate response close to 100% (17).

Recent advancements in targeted therapies have opened new avenues for treatment. Monoclonal antibodies such as daratumumab, which target CD38 on plasma cells, have shown exceptional activity in AL amyloidosis (18). This represents a significant step forward in the management of systemic AL amyloidosis, including those with GI involvement.

For ATTR amyloidosis, which was found in a subset of patients in recent studies, novel therapies such as patisiran and inotersen have been approved to slow the production of the abnormal TTR protein (9). These advances highlight the significance of precise typing in guiding treatment decisions.

The systemic character of amyloidosis, particularly the prevalence of cardiac involvement in AL and ATTR types, warrants a thorough assessment of individuals diagnosed with GIA. According to studies, up to 83.5% of AL and 100% of ATTR patients with GI involvement developed cardiac amyloidosis (5). This emphasizes the importance of using a multidisciplinary strategy to manage these individuals.

The importance of endoscopy in diagnosing GIA cannot be overemphasized. The duodenum has the best diagnostic yield for biopsy specimens, followed by the stomach, colon, rectum, and esophagus (14). This information can help physicians select the best biopsy locations for endoscopic treatments.

Despite the expansion of treatment options for GIA, barriers persist. The condition’s rarity, along with its nondescript appearance, continues to make diagnosis problematic. Furthermore, symptom control, especially in advanced cases, remains an important aspect of patient care. Supportive therapies for diarrhea, malnutrition, and other gastrointestinal problems are critical to improving quality of life (19).

## Conclusion

When patients are evaluated for unexplained, persistent epigastric pain and weight loss, it is prudent to rule out gastrointestinal amyloidosis as a possibility. Careful use of endoscopy with biopsy, along with advanced protein studies and cardiac imaging, can help confirm the diagnosis of GI amyloidosis and enable early recognition of heart involvement in the patient.

Diagnostic advances, notably proteomics-based typing, and the development of tailored therapies provide hope for a better prognosis. However, early detection is crucial, and a high index of suspicion is essential for prompt diagnosis and management. Future research is needed to identify more sensitive diagnostic tools and innovative therapeutic approaches to combat this difficult ailment. A multidisciplinary team approach is required to ensure proper diagnosis and to achieve a favorable patient outcome.

## Data Availability

The raw data supporting the conclusions of this article will be made available by the authors, without undue reservation.

## References

[1] Rowe K Pankow J Nehme F Salyers W . Gastrointestinal amyloidosis: review of the literature. Cureus. (2017) 9(5):e1228. doi: 10.7759/cureus.1228. PMID: 28611935 PMC5464793

[2] Inayat F Ur Rahman A Zahid E Ali NS Charles R . Symptomatic involvement of the stomach and duodenum as initial presentation of AL amyloidosis. BMJ Case Rep CP. (2019) 12(1):bcr–2018–227550. doi: 10.1136/bcr-2018-227550. PMID: 30659008 PMC6340590

[3] Song JH Jung HM Kim JW Kim ER Lee GY Yoon SE . Endoscopic features of gastrointestinal amyloidosis: a proposed endoscopic classification. Gut Liver. (2025) 19(4):592. doi: 10.5009/gnl240383. PMID: 40169391 PMC12261126

[4] Talar-Wojnarowska R Jamroziak K . Intestinal amyloidosis: clinical manifestations and diagnostic challenge. Adv Clin Exp Med. (2021) 30:563–70. doi: 10.17219/acem/133521. PMID: 33974753

[5] Hagen CE Dasari S Theis JD Rech KL Dao LN Howard MT . Gastrointestinal amyloidosis: an often unexpected finding with systemic implications. Hum Pathol. (2023) 139:27–36. doi: 10.1016/j.humpath.2023.06.007. PMID: 37390975

[6] Lekpa FK Ndongo S Pouye A Tiendrebeogo JW Ndao AC Ka MM . Amyloidosis in sub-saharan africa. Med Sante Trop. (2012) 22(3):275–8. doi: 10.1684/mst.2012.0085. PMID: 23174270

[7] Obici L Perfetti V Palladini G Moratti R Merlini G . Clinical aspects of systemic amyloid diseases. Biochim Biophys Acta (BBA)-Proteins Proteomics. (2005) 1753(1):11–22. doi: 10.1016/j.bbapap.2005.08.014. PMID: 16198646

[8] Cappello M Barbara G Bellini M Consalvo D Di Sabatino A Marasco G . Identification and management of gastrointestinal manifestations of hereditary transthyretin amyloidosis: Recommendations from an Italian group of experts. Digestive Liver Dis. (2024) 56(6):1014–1020. doi: 10.1016/j.dld.2023.11.025. PMID: 38105149

[9] Nakov R Suhr OB Ianiro G Kupcinskas J Segal JP Dumitrascu DL . Recommendations for the diagnosis and management of transthyretin amyloidosis with gastrointestinal manifestations. Eur J Gastroenterol Hepatol. (2021) 33(5):613–622. doi: 10.1097/meg.0000000000002030. PMID: 33394808

[10] Dahiya DS Kichloo A Singh J Albosta M Wani F . Gastrointestinal amyloidosis: a focused review. World J Gastrointest Endosc. (2021) 13(1):1–12. doi: 10.4253/wjge.v13.i1.1. PMID: 33520102 PMC7809597

[11] Sanchorawala V . Systemic light chain amyloidosis. N Engl J Med. (2024) 390:2295–307. doi: 10.1056/nejmra2304088. PMID: 38924733

[12] Muchtar E Dispenzieri A Magen H Grogan M Mauermann M McPhail ED . Systemic amyloidosis from A (AA) to T (ATTR): a review. J Internal Med. (2021) 289(3):268–292. doi: 10.1111/joim.13169. PMID: 32929754

[13] Fernandes DA Assis-Mendoça GR Costa LBED Freitas LLL Boin IFFS . Amyloidosis: a rare cause of severe acute liver failure. Rev Esp Enferm Dig. (2024) 116(1):44–45. doi: 10.17235/reed.2023.9598/2023. PMID: 36975149

[14] Cowan AJ Skinner M Seldin DC Berk JL Lichtenstein DR O'Hara CJ . Amyloidosis of the gastrointestinal tract: a 13-year, single-center, referral experience. Haematologica. (2013) 98(1):141–6. doi: 10.3324/haematol.2012.068155. PMID: 22733017 PMC3533676

[15] Poullos PD Stollman N . Gastrointestinal amyloidosis: approach to treatment. Curr Treat Options Gastroenterol. (2003) 6:17–25. doi: 10.1007/s11938-003-0029-2. PMID: 12521568

[16] Dobson CM . The structural basis of protein folding and its links with human disease. Philos Trans R Soc Lond B Biol Sci. (2001) 356:133–45. doi: 10.1098/rstb.2000.0758. PMID: 11260793 PMC1088418

[17] Malone MAV Castillo DAA Santos HT Kaur A Elrafei T Steinberg L . A systematic review of the literature on localized gastrointestinal tract amyloidosis: Presentation, management and outcomes. Eur J Haematol. (2024) 113(4):400–415. doi: 10.1111/ejh.14269. PMID: 39030954

[18] Roccatello D Fenoglio R Sciascia S Naretto C Rossi D Ferro M . CD38 and anti-CD38 monoclonal antibodies in AL amyloidosis: targeting plasma cells and beyond. Int J Mol Sci. (2020) 21(11):4129. doi: 10.3390/ijms21114129. PMID: 32531894 PMC7312896

[19] Fritz CD Blaney E . Evaluation and management strategies for GI involvement with amyloidosis. Am J Med. (2022) 135:S20–3. doi: 10.1016/j.amjmed.2022.01.008. PMID: 35077702

